# Smooth muscle cells from skin perforator vessels as a new tool for vascular research

**DOI:** 10.1007/s10974-025-09704-z

**Published:** 2025-07-31

**Authors:** Alisah Hussain, Yaw Asare-Amankwah, Nikolaos S. Lymperopoulos, M. Julie Thornton, Kirsten Riches-Suman

**Affiliations:** 1 Cardiovascular Research Group, University of Bradford, Bradford, West Yorkshire UK; 2 Plastic Surgery Department, Pinderfields Hospital, the Mid-Yorkshire NHS Trust, Wakefield, West Yorkshire UK; 3 Centre for Skin Sciences, University of Bradford, Bradford, West Yorkshire UK

**Keywords:** Vascular smooth muscle cell, Cell phenotype, Perforator vessels, Cell morphology, Proliferation

## Abstract

Vascular smooth muscle cells (SMC) comprise the medial layer of blood vessels and are responsible for maintaining vascular tone. Ordinarily quiescent and contractile, SMC can dedifferentiate into different phenotypes following injury or in disease states such as atherosclerosis and are thus valuable research tools for examining these conditions. The isolation of commonly used SMC types, such as those from the aorta or saphenous vein (SV), require clinical links or commercial supply and are rarely pathology-free. The skin is highly vascularised with perforator (Perf) vessels that protrude through the skin layers to feed the tissue. Whilst these vessels can be sourced from clinical procedures (e.g. reconstructive surgery), they are also available from elective cosmetic procedures such as abdominoplasty which could provide blood vessels unaffected by an underlying pathology. This paper describes the isolation of Perf-SMC for the first time, using a cost-effective explant technique. Explanted cells were confirmed as SMC by co-staining for alpha smooth muscle actin and smooth muscle myosin heavy chain. Phenotypic characteristics of Perf-SMC (cell morphology, proliferation, and multinucleation) were comparable to those from commonly used SMC from alternative vascular sources (SV-SMC and umbilical artery SMC). Furthermore, Perf-SMC were stable in culture up until at least passage 9 with no alteration in morphological characteristics or evidence of replication-induced phenotypic change. In summary, this paper describes an effective, efficient and low-cost method for isolating SMC from skin perforator vessels that may be a useful tool for the future examination of SMC behaviour from both pathological and healthy skin.

## Introduction

Vascular smooth muscle cells (SMC) are the principal cell type in the vascular wall. In comprising the medial layer of all blood vessels, their main role is in contraction and relaxation to maintain vascular tone. SMC are not terminally differentiated and are able to switch their phenotype according to external cues. Whilst ordinarily quiescent with a low turnover rate and high expression of contractile machinery, they can transiently and reversibly switch to a synthetic phenotype. This is characterised by proliferation and secretion of inflammatory cytokines and growth factors. Whilst this is beneficial when blood vessels need to respond to injury, if it persists for longer than needed or occurs in the absence of injury, it can lead to the development of cardiovascular diseases such as atherosclerosis or neointimal hyperplasia (Owens et al. 2004). Furthermore, understanding of the diversity of potential SMC phenotype(s) has expanded considerably over the past decade, with SMC being able to adopt osteogenic, macrophage-like, mesenchymal stem cell-like and myofibroblast-like phenotypes in addition to the traditionally recognised contractile and synthetic phenotypes (Riches-Suman and Hussain 2022). Because of this plasticity, SMC are commonly used in mechanistic studies into the molecular and cellular causes underpinning vascular disease.

Many experiments are performed on vascular SMC purchased from commercial sources. These are valuable in that the cells are isolated from vessels most commonly involved in cardiovascular morbidities, for example the aorta, pulmonary artery or coronary arteries. Additionally, the use of commercial SMC negates the need for researchers to have clinical collaborations for the provision of fresh tissue. The highly plastic nature of SMC underscores the importance of using cells from more than one patient donor to fully evaluate biological mechanisms; however, purchasing multiple vials from different patient donors with proprietary growth media compositions can become financially prohibitive. Furthermore, there can be potential import restrictions or delays that impact on the availability of these cells.

One way to assess inter-donor variability is to establish links with clinical collaborators that allows the explant of SMC that are surplus to surgical procedures. Examples include the saphenous vein (SV) and internal thoracic artery. These are removed during coronary artery bypass graft surgery and surplus fragments are commonly used to grow SMC (Yang et al. 1998). Collaboration with cardiac surgeons in this instance can provide a regular supply of tissue from multiple patients where SMC behaviour can be assessed in vitro. However, this SMC supply requires invasive and medically-necessary surgery which can make finding ‘healthy’ control SMC difficult as all patients undergoing surgery have some type of cardiovascular pathology.

**Fig. 1:**
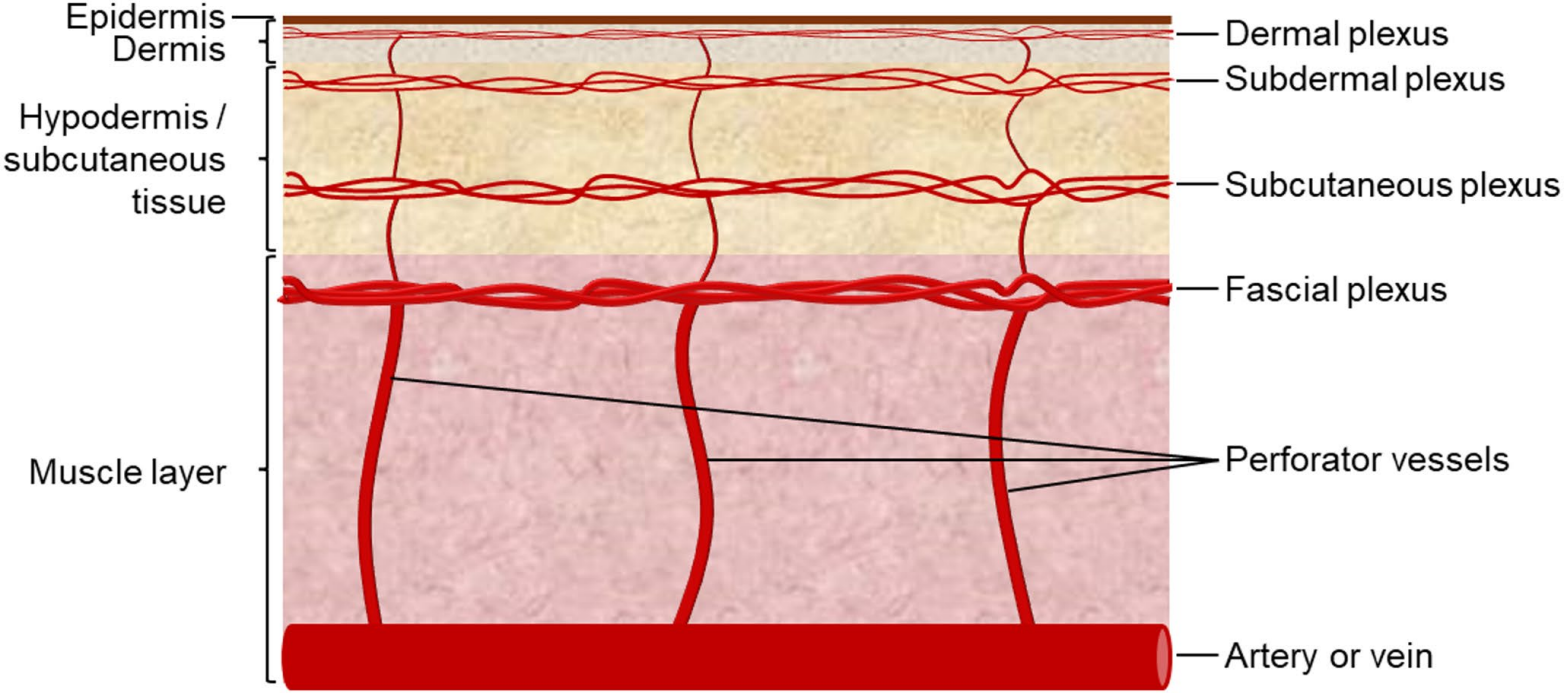
Simplified schematic of the skin vasculature. Human skin is fed from a rich vascular network. Perforator vessels project outwards from arteries or veins to feed the fascial, subcutaneous, subdermal and dermal plexuses; passing through all layers of the skin with the exception of the avascular epidermis

The skin is the largest organ in the human body and is highly vascularised (Fig. 1). Perforator vessels project from arteries or veins in the muscle layer to feed the subcutaneous and dermal tissues, ensuring a consistent blood flow to and from the skin (Saint-Cyr et al. 2009). They are integral to reconstructive surgeries that utilise a flap such as post-mastectomy breast reconstruction, or head and neck reconstruction (Lyons 2006). Whilst fragments of these perforating vessels are available from these clinical reconstructive surgeries, they are also amply available from tissue typically removed through amputation or elective cosmetic surgeries such as abdominoplasty or reduction mammoplasty. These tissues are usually discarded as clinical waste and thus could represent a rich vascular resource to allow SMC isolation from both pathological (clinical) and non-pathological (elective) samples across multiple donors.

This paper describes for the first time a low cost, reproducible and robust methodology for isolating SMC from perforator vessels of the leg. It describes the method for locating the vessels within the tissue and their dissection to reliably obtain SMC cultures. Furthermore, the in vitro SMC phenotype is evaluated in comparison to other vascular SMC sources, and the stability of the phenotype across multiple passages in culture is measured.

## Materials and methods

### Tissue collection and SMC isolation

Samples of human skin were collected from patients undergoing lower limb amputation at the Bradford Royal Infirmary. All donors gave written informed consent prior to their inclusion in the study. The study had local ethical committee approval (16/077) and conformed to the ethical standards laid down in the declaration of Helsinki.

Tissue was laid epidermis-down in a 10 cm^2^ petri dish and perforating vessels projecting into the subcutaneous tissue were identified and dissected out from the surrounding tissue using sterile fine forceps and a scalpel blade. The vessel was removed to a 5.5 cm^2^ petri dish and covered with growth media (Dulbecco’s Minimal Essential Medium (DMEM) with 4.5 g/L glucose, 1% (v/v) penicillin-streptomycin-fungizone, 1% (v/v) L-glutamine, 10% (v/v) fetal bovine serum). Any adventitial material was delicately removed using the blade, and the vessels opened longitudinally using micro-dissecting scissors. The endothelial layer was gently removed by scraping. The vessel was then cut into ~ 1 cm lengths. Each length was transferred to a new 5.5 cm^2^ petri dish, covered with a few drops of growth media, and cut into 1–2 mm^2^ cubes. These were resuspended in 2 mL of growth media and transferred to a 25 cm^2^ flask. These were left undisturbed in a tissue culture incubator at 37^o^C in 5% CO_2_ in air for one week. After this time, cells were seen explanting out from the tissue. Growth media (0.5 mL) was added twice per week until the volume in the flask reached 5 mL; after this time cells underwent a half media change twice per week until ~ 80% confluent. At this point, cells were passaged using trypsin-EDTA into a 75 cm^2^ flask. Growth media was half changed twice per week and cells were sub-cultured with a 1:3 split when they reached ~ 80% confluence.

SV-SMC were isolated as previously described (Porter et al. 2002). Umbilical artery (UA)-SMC were sourced from Ethical Tissue (Bradford). All SMC cell types were cultured in the same growth media as above under identical conditions. Summary donor characteristics can be found in Table 1.

**Table 1:** Tissue donor demographics. Summary information on the gender, age and ethnicity of patients who donated tissue for smooth muscle cell explant

Vessel type	Gender (M: F)	Age (mean; range)	Ethnicity
Perforator	2:1	69; 64–73 years	Caucasian
Umbilical artery	0:3	26; 22–31 years	1:2 South Asian: Caucasian
Saphenous vein	2:1	34; 31–39 years	1:2 South Asian: Caucasian

### Immunocytochemistry

SMC were seeded onto glass coverslips in 24-well plates at a density of 10,000 cells per well. After 72 hours, they were fixed in 4% (w/v) paraformaldehyde for 12 minutes. Coverslips were washed with PBS three times and then permeabilised with 0.1% (v/v) Triton X-100 in PBS for 30 minutes. Non-specific binding was blocked using 0.1% (v/v) Tween-20 in PBS supplemented with 5% (w/v) bovine serum albumin for 15 minutes. Primary antibodies (1:250 rabbit anti-smooth muscle myosin heavy chain [EPR5336(B)] – SM-MHC; 1:250 mouse alpha smooth muscle actin [1A4] – α-SMA; and 1:100 rabbit phospho-histone H2AX [Ser139] - γH2AX; all sourced from AbCam) were diluted in 0.1% (v/v) Tween-20 in PBS. Cells were incubated with primary antibodies overnight at 4^o^C in a humidified chamber. After this, coverslips were washed three times with 0.1% (v/v) Tween-20 in PBS supplemented with 5% (w/v) BSA. Secondary antibodies (Alexa-fluor 568 F(ab’) fragment goat anti-rabbit IgG (H + L) and Alexa-fluor 488 F(ab’) fragment goat anti-mouse IgG (H + L); both ThermoFisher Scientific) were diluted 1:500 in 0.1% (v/v) Tween-20 in PBS. Cells were incubated with secondary antibodies for 4 h at room temperature, protected from light. Coverslips were then washed three times in PBS and mounted onto glass slides using ProLong Gold with DAPI nuclear stain. Labelled cells were imaged at x200 magnification. Five random fields of view were captured for each coverslip.

### Morphometrics

Five random fields of view of subconfluent proliferating SMC were imaged at x200 magnification. The area and circularity of fifty cells per donor were measured using ImageJ software version 1.53 as previously described (Al-Rikabi et al. 2021).

### Proliferation

Cells were seeded at a density of 10,000 cells per well in 24-well plates. Cells were allowed to attach overnight and then transferred into serum-free growth media for 48 h. Day 0 cell counts were taken and the remaining cells stimulated with growth media containing 10% (v/v) fetal bovine serum for up to one week. On days 2, 4 and 7, counts were taken and remaining cells re-treated with growth media. Cells were counted using trypan blue exclusion from triplicate wells at all time points.

### Nuclear characteristics

Nuclear characteristics were measured as described previously (Riches et al. 2018). Briefly, the number of nuclei per cell was evaluated in ~ 50 cells per donor using the light microscopy images that were acquired for cell morphometry measurements. The morphology of the nuclei in ~ 50 cells per donor was evaluated from the images captured for fluorescence microscopy and classified as ‘normal’ (ovoid with smooth boundary) or ‘aberrant’ (irregularly shaped, blebbed, invaginated). The presence of γH2AX-positive foci was quantified as the proportion of nuclei that had at least one foci evident relative to the total number of nuclei.

### Statistics

All data are presented as mean ± standard error of the mean, and *n* refers to the number of individual patient donors. Morphological and DNA damage measurements were analysed using Kruskal-Wallis test with Dunn’s post-hoc test or Wilcoxon matched-pairs signed rank test as appropriate. Proliferation data was analysed using an ordinary 2-way ANOVA with Tukey’s multiple comparison test.

## Results

### Identification of blood vessels and isolation of SMC

Tissue was received from three individual donors. These varied in terms of absolute size, yet appreciable vessels were identified in all of them with similar cross-sectional diameters of ~ 1 mm. We found the easiest way to identify the perforator (Perf) blood vessels was to look around the cut border of the tissue within the subcutaneous fat to find an appreciable lumen. If no lumen was visible, sequential full thickness cuts were made in the tissue, working in from the borders a few millimetres at a time, until a lumen could be seen. Once the lumen was identified, it was secured using fine forceps and the tissue surrounding the vessel gradually dissected away using a scalpel blade to reveal the length of the vessel.

**Fig. 2:**
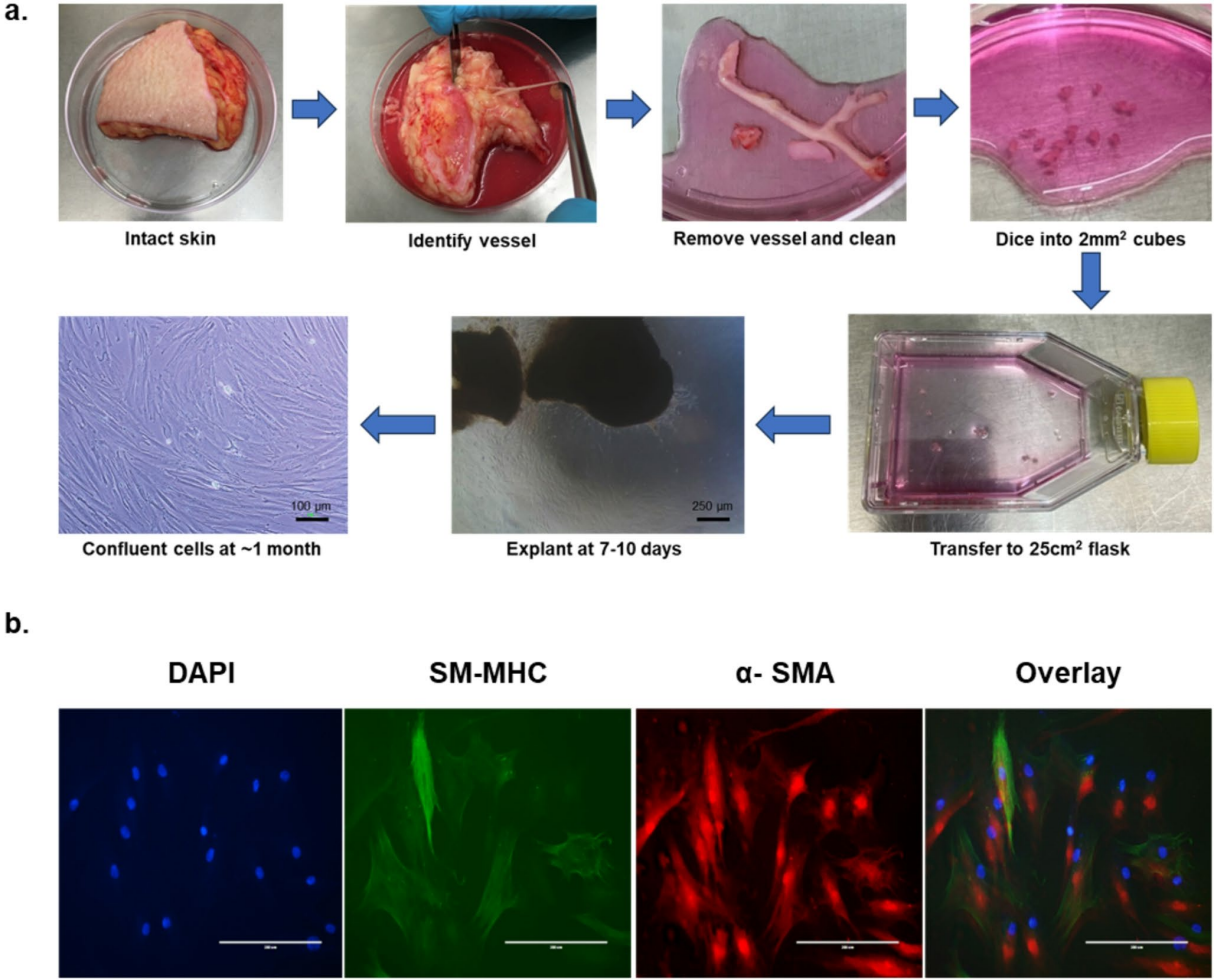
Isolation and validation of perforator smooth muscle cell identity. (a) Human skin tissue was collected from the lower limb of patients undergoing amputation. The skin was dissected, dermis side up, and the perforator vessels identified and removed. These were covered in growth media and the adventitia was dissected off. The vessels were opened longitudinally, the endothelial layer was scraped away, and the resulting medial tissue was dissected into ~ 2 mm^3^ pieces. These were transferred to a 25 cm^2^ tissue culture flask and maintained in a tissue culture incubator. After approximately 1 week, cells could be seen migrating from the edges of the tissue and the flask was confluent within 1 month. (b) The identity of the cells as smooth muscle cells was confirmed using co-staining for smooth muscle myosin heavy chain (SM-MHC), alpha smooth muscle actin (α-SMA) and DAPI nuclear stain at passage 1. Scale bar = 200 μm; images are representative of *n* = 3 independent isolations

The length of vessel that was isolated from each tissue sample varied between 1 and 3 cm. Once removed from the tissue bed, the adventitia was removed using fine forceps and a scalpel. Despite the small lumen size, vessels were able to be opened longitudinally and gently scraped to remove the endothelium – care was taken here not to apply too much pressure that the vessel was cut. Irrespective of the starting length, the opened vessels were cut into smaller ~ 1 cm lengths. Each of these was cut into 1–2 mm^3^ cubes and put into separate flasks. There was no difference in the speed of explant between the donated tissue samples, with SMC becoming visible within 1 week of explant and confluency being reached within 4–6 weeks (Fig. 2a).

Following explant, the identity of the cells was verified using co-staining for SM-MHC (a SMC marker protein) and α-SMA; a protein enriched in SMC but that can also be expressed in other cell types including myofibroblasts (Owens et al. 2004). All cells stained positively, confirming a pure population of SMC that was not contaminated by other vascular cell types such as endothelial or adventitial fibroblast cells (Fig. 2b).

### Comparison of SMC phenotype with SMC from other commonly used tissues

After confirming that the explanted cells were SMC, we wanted to compare their appearance and function with SMC from alternative sources that have already been characterised. For this, we chose the umbilical artery (UA) as an alternative arterial source, and saphenous vein (SV) as an alternative venous source. We have extensive experience in using and characterising these cells (Hemmings et al. 2021; Riches et al. 2014a, b).

**Fig. 3:**
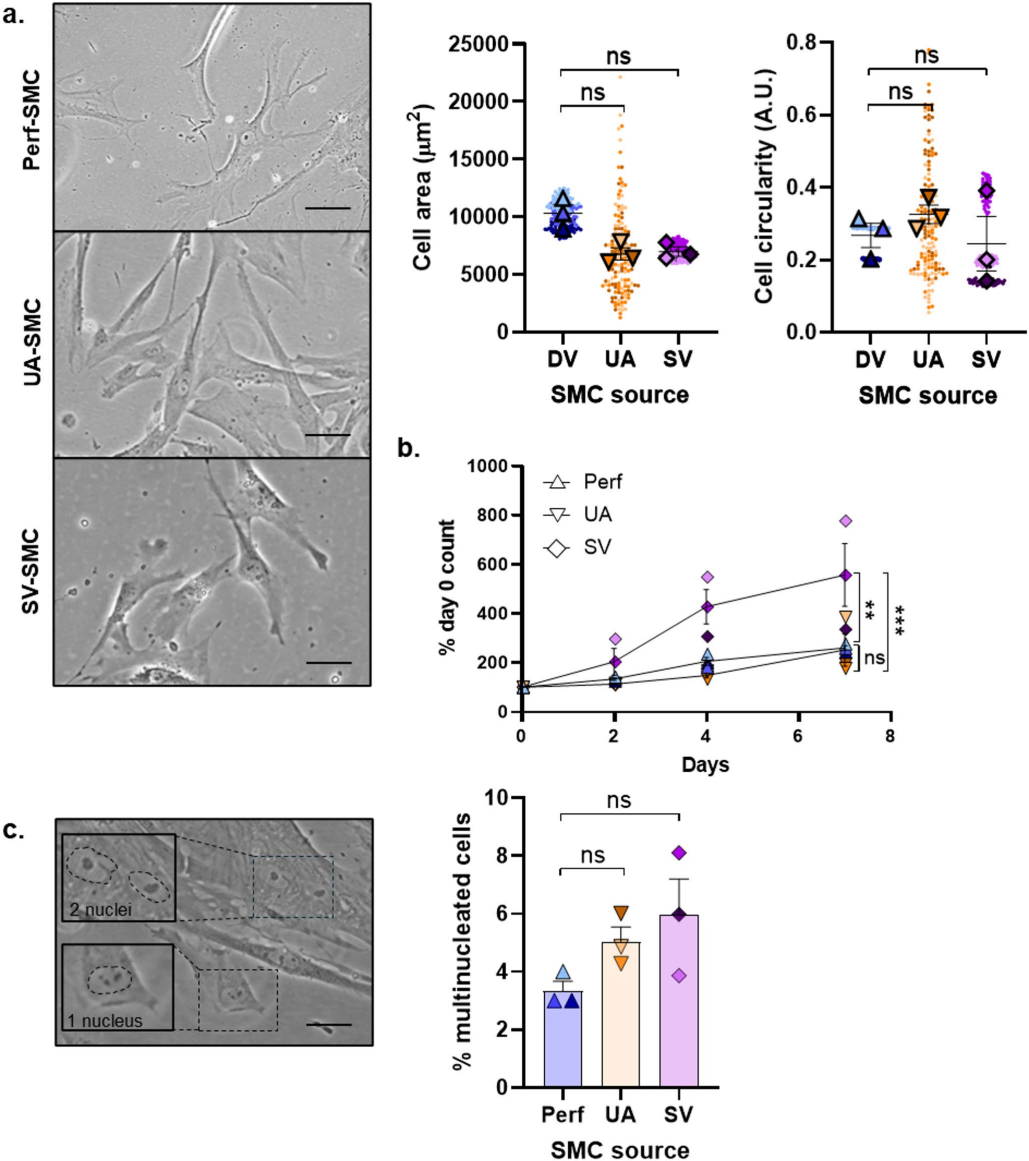
Characterisation of Perf-SMC phenotype compared to other vascular SMC sources. (a) The area and circularity were measured from fifty proliferating cells per patient donor of SMC isolated from the skin perforator (Perf), umbilical artery (UA) or saphenous vein (SV). Scale bar = 50 μm. (b) The proliferation of SMC from all sources were measured in full growth media over 7 days. (c) The proportion of multinucleated cells in five fields of view per patient donor were quantified from all SMC sources. Scale bar = 50 μm. Insets show an example of mono- or bi-nucleated cells. ***P < 0.001, **P < 0.01, ns = not significant. *n* = 3 patient donors for each cell source. Small circles represent individual cells, and larger triangles or diamonds represent the summary data. Each donor is presented as a separate colour

The spread cell area of Perf-SMC was 10,295 ± 764 µm^2^. Although larger than UA-SMC (6,092 ± 930 µm^2^) and SV-SMC (6,994 ± 407 µm^2^), the difference was not significant. The spindle morphology of the cells was also consistent across SMC from each tissue type with no difference in average circularity (Fig. 3a). SMC function was assessed using 7-day proliferation curves. Over the course of 7 days, Perf-SMC increased their cell number by 3.3-fold. This was similar to UA-SMC, but less than SV-SMC proliferation rates where cell number increased by 5.6-fold. This was significantly higher than both Perf- and UA-SMC (Fig. 3b).

We also evaluated the stress of the SMC by looking at the proportion of multinucleated cells as a marker of potential DNA damage. Only 3.33 ± 0.33% of Perf-SMC exhibited more than one nucleus per cell. UA-SMC were slightly higher at 5.04 ± 0.51%, and SV-SMC higher still at 5.98 ± 1.22%. However, there was no significant difference between the source tissue types (Fig. 3c). From this data, we have shown that Perf-SMC have a comparable in vitro cell morphology, proliferative capacity and multinucleation to UA-SMC and, with the exception of proliferative rate, SV-SMC.

### Maintenance of phenotype through passaging

SMC are not terminally differentiated, and other studies have suggested that primary SMC can lose their phenotypic characteristics as early as passage 7 (Chang et al. 2014). Thus, once we had established that Perf-SMC were phenotypically comparable to more commonly used SMC, we were interested to see how long they maintained their phenotype in culture. To do this, we serially-passaged the Perf-SMC and compared the morphological, proliferative, and stress-related characteristics at low passage (p2-3) and at high passage (p8-9).

**Fig. 4:**
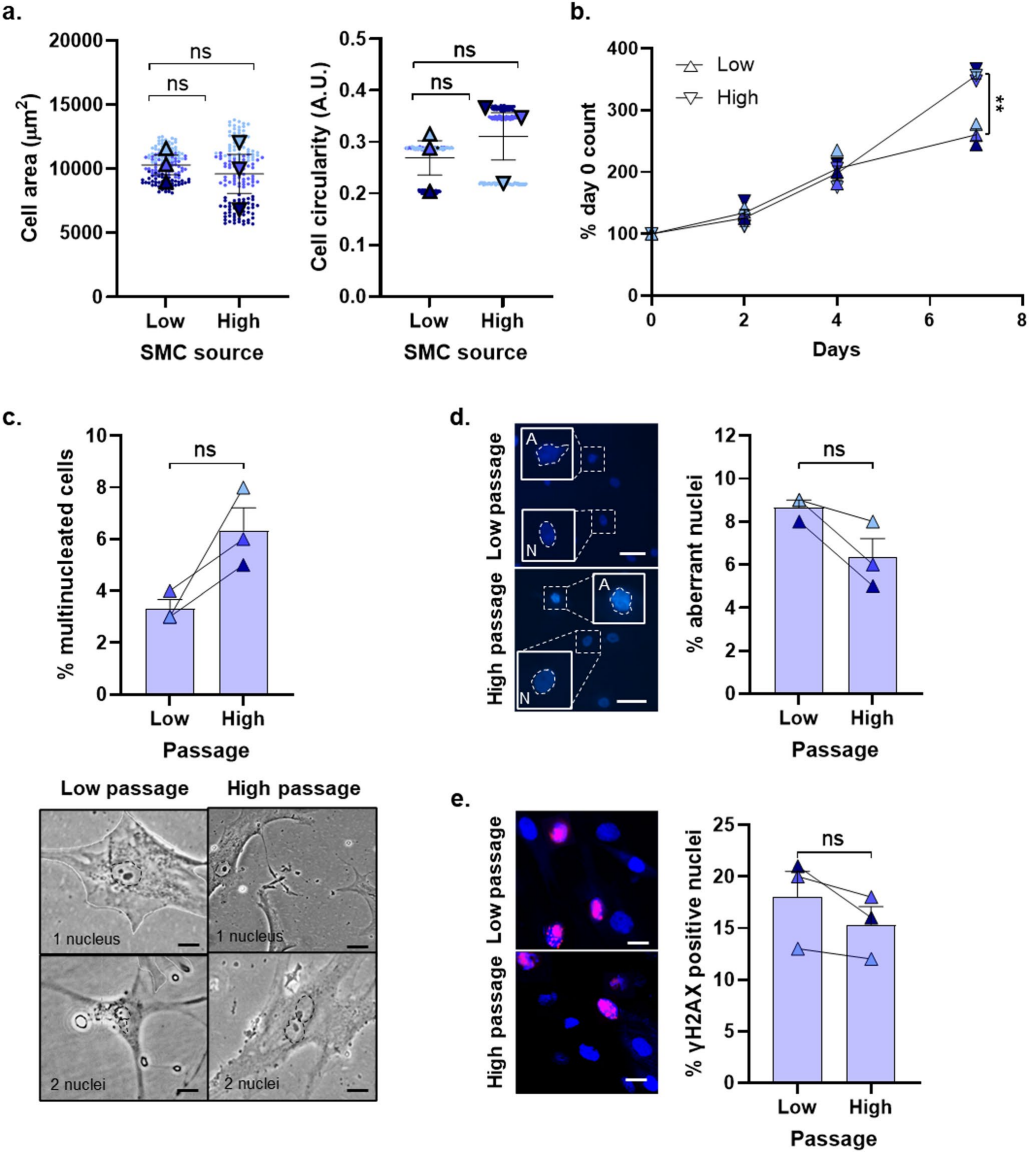
Stability of Perf-SMC phenotype across passages. Matched Perf-SMC were cultured and their parameters measured at both low (p2-3) and high (p8-9) passages. **a** Area and circularity from fifty proliferating cells per patient donor. **b** Proliferation in full growth media over 7 days. **c** The proportion of multinucleated cells (scale bar = 20 μm), **d** aberrantly-shaped nuclei (scale bar = 50 μm; insets show examples of aberrant ‘A’ and normal ‘N’ nuclear morphology using DAPI staining) and **e** γH2AX-positive nuclei (scale bar = 20 μm; insets show γH2AX as pink foci on DAPI-stained nuclei) was quantified in five fields of view per patient donor for matched low and high passage Perf-SMC. **P < 0.01, ns = not significant. *n* = 3 patient donors for each cell source. Small circles represent individual cells, and larger triangles represent the summary data. Each Perf-SMC donor is presented as a separate colour shade

The appearance of Perf-SMC was not different between low and high passage, with comparable cell areas and circularity in both conditions (Fig. 4a). Moreover, the proliferative capacity of low and high passage cells was comparable at early timepoints but was significantly higher for high passage cells at day 7 (Fig. 4b). There was almost double the number of multinucleated cells in high passage SMC compared to low passage SMC (Fig. 4c). This did not reach statistical significance, however we decided to further interrogate markers of cell stress and DNA damage, to confirm or negate the suggestion of increased stress.

The nuclei in cells which have undergone DNA damage or premature in vitro ageing can become deformed (Hänzelmann et al. 2015) and so we quantified the proportion of cells that had an irregularly shaped nucleus. For this, we considered ovoid or round nuclei to be ‘normal’ and any nuclei that had clefts, invaginations, non-ovoid shapes or evidence of micronuclei adjacent to the main nucleus to be ‘aberrant’. At low passage, 8.67 ± 0.33% of cells had aberrant nuclei, and this was similar at high passage which had a frequency of 6.33 ± 0.88% SMC (Fig. 4d). We also looked more deeply at a sub-organelle level and performed immunocytochemistry for γH2AX, which is a marker of double-stranded DNA breaks (Valdiglesias et al. 2013). At low passage, 18.00 ± 2.50% of SMC exhibited at least one γH2AX-positive loci in their nucleus. At high passage, this value was not significantly different at 15.33 ± 1.76% of SMC (Fig. 4e).

Thus, although there was a suggestion of increased DNA damage according to the proportion of multinucleated cells, this was not borne out by further interrogation using different assays. From this, we conclude that the morphology of Perf-SMC is stable across passage up to at least passage 9, and that they are not undergoing replicative senescence or an increased rate of DNA damage and stress at these later passages.

## Discussion

As the principal cell type in the vascular wall, SMC are critical in maintaining the function of healthy blood vessels and are implicated in the initiation and progression of many vascular disorders including atherosclerosis, neointimal hyperplasia and hypertension, amongst others. SMC are known to be phenotypically diverse, and behaviour can differ according to vascular bed (Majesky 2007; Owens et al. 2004). SMC phenotype also differs from person-to-person, and so any investigations into SMC behaviour need to consider this inherent diversity. Many studies on human SMC use commercially sourced SMC with a limited number of patient donors and the costs associated with increasing these *n* numbers can be prohibitive. The skin is a highly vascularised organ, with surplus skin commonly removed during necessary and/or elective procedures (for example, amputations, tissue reconstruction, mammaplasty, abdominoplasty). This is ordinarily discarded. Our method provides a reliable, cost-effective way to isolate primary human SMC from this surplus tissue. This opens avenues to research new types of vasculature and increase the number of patient donors from both ‘healthy’ control tissues and from cardiovascular pathologies, altogether giving a more robust assessment of phenotypic behaviour and plasticity in a diverse population.

Perf-SMC were successfully explanted from all patient donors. Moreover, the time taken to start to explant was approximately one week, with confluence gained within 4–6 weeks. This is comparable to explant speeds from SV tissue where cells were visible within 1–2 weeks and were ready to passage at 5–6 weeks (Porter et al. 2002). Human UA-SMC reportedly have faster explant speeds (3–4 days) and reach confluence more quickly with some as soon as 2 weeks after explant (Martín de Llano et al. 2007), however these explants were conducted on collagen-coated dishes which may have help promote more rapid migration and proliferation of resident SMCs. This demonstrates that the explant method on standard plastic tissue culture dishes is just as effective for Perf-SMC as it is for other commonly-used SMC types, and generates a pure population of SMC free from endothelial or adventitial fibroblast contamination.

SMC exhibit a remarkable degree of plasticity where they can exist in contractile spindle-shaped or synthetic rhomboid-shaped phenotypes, and anywhere in between (Owens et al. 2004). Morphological measurements including the area of the cell are useful for assessing changes in the behaviour of SMC (Wang et al. 2023) and can be used as an indicator of phenotype with larger, rounder cells being indicative of a proliferative synthetic phenotype and smaller, spindle-shaped cells representing a contractile phenotype (Thakar et al. 2009). We have previously measured the spread cell area of SV-SMC and found the mean area to be between 4 and 6,000 µm^2^ (Riches et al. 2013, 2014a, b). This is comparable to what we see in the current study. Perf-SMC measured in parallel to SV-SMC were consistently larger at an average size of 10,295 µm^2^, however this was not significantly different. Instead, it was more comparable to the mean area of alternative arterial SMC such as those from the internal thoracic artery (9,586 µm^2^; (Riches et al. 2014b). Interestingly, the circularity of the cells was almost identical between Perf- and SV-SMC, suggesting that although Perf-SMC may be slightly larger, they had not dedifferentiated into a rhomboid phenotype.

Growth curves over 7 days revealed that Perf-SMC and UA-SMC had similar proliferation rates which were similar to observations in other arterial SMC types such as internal thoracic artery. SV-SMC proliferated much more quickly (5.6-fold) which is in keeping with previous studies (Riches et al. 2014b; Turner et al. 2007). It has been previously shown that venous SMC proliferate more quickly than arterial SMC in both human and animal models (Wong et al. 2005; Yang et al. 1998). Based on this, plus the similarity of Perf-SMC morphology with internal thoracic artery SMC, we believe that the perforator vessels isolated in this study were arterial in origin. All of this, in combination with the Perf-SMC showing comparably low levels of stress to SV- and UA-SMC, leads us to conclude that the dermal perforator SMC have a similar phenotype and function to alternative SMC sources and are a valuable addition to the cell types that are available to study vascular function in vitro.

One of the main challenges of using primary cells in culture is their tendency to undergo replicative senescence. We have previously shown that SMC from internal thoracic artery and SV retain their morphological characteristics up until at least passage 6 (Riches et al. 2014b). Other studies on human coronary artery or aortic SMC reveal phenotypic changes at ‘high’ passages, but what is considered as ‘high’ varies amongst studies. Whilst some consider passages of 5 and 6 to be low and passage 11–13 to be high (Bielak-Zmijewska et al. 2014; Nakano-Kurimoto et al. 2009), others consider passage 2–3 to be low with phenotypic loss evident as soon as passages 6–8 (Chang et al. 2014; Stojanović et al. 2020). For our assessment of phenotypic stability, we elected to examine matched cells at passage 2–3 (low) and passage 8–9 (high).

We found that the morphology of SMC did not change between these passage points, both in terms of absolute cell area and in terms of spindle morphology. Enlargement of SMC undergoing replicative senescence is well established (Stojanović et al. 2020), and so we are confident that Perf-SMC do not undergo replicative senescence or phenotype loss by passage 9. Replicative senescence is associated with permanent withdrawal from the cell cycle (Chandler and Peters 2013) and thus reduced proliferative capacity; again our data showing that the proliferation of Perf-SMC was actually greater at high passage strengthens our assertion that Perf-SMC are not undergoing replicative senescence. However, it could suggest a transition to a synthetic phenotype. Given that the morphology of the cells is unaltered, further studies examining synthetic behaviours at these later passages would be warranted in order to make any conclusions regarding a synthetic phenotypic switch. The number of nuclei per cell was slightly (but not significantly) greater for high passage cells. To further examine this, we assessed other markers of DNA damage such as changes in nuclear morphology or double-stranded DNA breaks which are evident in cells following replicative senescence (Bielak-Zmijewska et al. 2014; Hänzelmann et al. 2015). We confirmed there was no evidence of increased DNA damage at higher passages and thus are confident that Perf-SMC can be used for in vitro studies of vascular function up until at least passage 9. In the future, assessing other behavioural and phenotypic features such as responses to contractile agonists, organisation of the cytoskeleton, and quantitative assessment of contractile markers such as calponin or smoothelin would be beneficial to fully evaluate Perf-SMC alongside other commonly used SMC types.

In summary, we believe this to be the first report of successfully isolating and characterising SMC from a new source—perforating vessels that feed the skin. SMC were explanted on tissue culture plastic with no need for pre-coating with gelatin, collagen or fibronectin. The Perf-SMC were comparable in appearance and function to other commonly used SMC types and were phenotypically stable in culture. This vascular SMC source may be more accessible for routine isolation due to elective surgeries such as cosmetic procedures, increasing the potential number of donors from which cells could be isolated. This would provide a much better representation of the inherent inter-patient variability in vascular biology. Whilst it remains to be seen whether there are phenotypic differences from different skin sites (for example, haired, non-haired, axillary skin), the method described here provides an ideal starting point for examining SMC phenotype from this new vascular source.

## Data Availability

The data that support the findings of this study are not openly available due to reasons of sensitivity and are available from the corresponding author upon reasonable request. Data are located in controlled access data storage at the University of Bradford.
